# The Treatment of Typhoid Fever Especially by Antiseptic Remedies

**Published:** 1892-03

**Authors:** 


*The Treatment of Typhoid Fever especially by Antiseptic Remedies.* By J. BURNEY YEO, M.D., F.R.C.P. Pp. 70. London: Cassell & Co. 1891.

In this little book much information is conveyed in moderate compass, and those who wish to learn the most improved methods of treatment of typhoid fever will get real help from it. Dr. Yeo fully considers the "antiseptic" treatment of typhoid, which has been brought into prominence of late. He gives an account of the various ways in which different physicians carry it out, with a description of his own plan of treatment, for the details of which the reader may be referred to the book itself. In Dr. Yeo's hands, as in those of other observers, this treatment has been attended with very good results, and as it gradually becomes perfected will we think more and more recommend itself as the most sound and rational mode of combating the disease.

